# Promethazine is not a good option to aid sleep quality, especially for people using psychiatric services

**DOI:** 10.1192/bjb.2024.108

**Published:** 2025-12

**Authors:** Jacob D. King

**Affiliations:** 1 Division of Psychiatry, Imperial College London, UK; 2 Central and North West London NHS Foundation Trust, London, UK

**Keywords:** Sleep–wake disorders, general adult psychiatry, mental health services, psychopharmacology, psychological treatments

## Abstract

Promethazine, a sedating antihistamine, is widely and increasingly prescribed for patients reporting problems sleeping. In this Against the Stream article, the case is made that promethazine is not suitable as a sleep aid for people using mental health services, because it has no good evidence base, impedes with psychological and behavioural techniques that do improve sleep in the medium-long term, has underappreciated addictive and recreational-use potential, and an unacceptable side-effect profile. Alternatives to promethazine are described, notably the NICE first-line recommendation, cognitive–behavioural therapy for insomnia.

Promethazine is a sedative phenothiazine compound, which has a long history in psychiatry, initially intended to treat psychosis, but which has now found a place for treating allergy, nausea and as a sedative agent. Described as a first-generation antihistamine, promethazine has strong affinity as an antagonist of H_1_ histaminergic receptors of the tuberomammillary nucleus, as well as moderate antagonism of muscarinic cholinergic receptors, and weak-moderate affinity to receptors in the serotonergic, dopaminergic, adrenergic and glutaminergic systems.

Despite being an older medication, there has been a sharp increase in use of promethazine. English General Practice prescribing data shows a doubling of prescriptions in the past 5 years alone, with 215 000 scripts written in August 2024 (https://openprescribing.net/chemical/0304010W0/). This trend holds true where promethazine was prescribed specifically as a sleep aid in mental health services; for example, in a Danish cohort,^1^ and in a local review of out-patient psychiatric prescribing within one of our community teams in London, promethazine 25 mg once nightly was the single most prescribed non-routine medication, with ‘for sleep’ the near unanimous indication (details available from the author on request).

Promethazine has most probably gained common usage in psychiatry because of its familiarity to prescribers, pharmacists and dispensers as part of rapid tranquilisation protocols. The first-generation antihistamines promethazine and diphenhydramine are also available over the counter in UK, which may create the impression that they are generally safe to use. Importantly, there is limited access internationally to the National Institute for Health and Care Excellence (NICE) guidelines’ first-line recommended treatment for insomnia, cognitive–behavioural therapy for insomnia (CBTi), and it is therefore entirely understandable that clinicians, who want to support their patients’ distress, will prescribe medication in place of unavailable CBTi. Moreover, the act of being prescribed and dispensed a medication itself may also have reassuring non-physiological, psychodynamic benefit for a patient.

It is important to highlight that although both are reduced states of consciousness, sedation is not sleep. These two brain states are different phenomenologically and physiologically, and appear different on electroencephalography. Many of the neurophysiological and psychological benefits of natural human sleep do not occur under other states of sedated consciousness, and many sedative agents increase sleep debt, are associated with neurotoxicity and contribute to non-restorative sleep.^2^ Prescribing a sedative agent for use in severe agitation or crisis episodes resulting from a mental illness, where supporting the dissociation – the withdrawal of the patient from an external world that is temporarily overwhelming while social, psychological, pharmacological or environmental changes are made – is quite different to aiding sleep quality in either the short or long term. There are apparent contradictions between the increasing use of promethazine to aid the sleep quality of people using psychiatric services, and that promethazine, for reasons that will be outlined, is an unsuitable agent for this intent.

In the first instance, we must bear in mind that there is no good-quality evidence that promethazine is effective in improving sleep quality.^3^ No clinical trials have been conducted, and only a handful of small, old, experimental non-controlled studies looking at promethazine outcomes on human sleep electroencephalography are published, demonstrating reduced sleep-onset latency and reduced percentage of rapid-eye movement sleep at doses of 40 mg.^4^ Moreover, H_1_ antagonism is a complex and poorly understood pharmacokinetic and pharmacodynamic process, which may be dependent on other endogenous sedating and wake-promoting systems, and means that we ought not expect to see predictable linear effects akin to GABAergic agents (i.e. benzodiazepines or Z-drugs).^5^ Although in the UK, promethazine is licenced by the MHRA for very-short-term use in insomnia disorder, it is not recommended by NICE insomnia guidelines.^3^ We must, therefore, be cautious in expecting any benefit to sleep quality, and remember that promethazine is not licensed as a treatment for any other sleep disorder.

## Concerns also relate to three further factors

First, the use of promethazine interferes directly with employing medium-long-term psychological and behavioural strategies that, unlike promethazine, have been proven to improve sleep quality. CBTi is effective among people with co-occurring mental state disorders,^6^ and has continued benefits demonstrated for as many as 10 years later.^7^ CBTi is a short-term psychological treatment that teaches people how to regulate their sleep–wake behaviour, strengthen the association of feeling sleepy and being in bed, and provides sleep psychoeducation and techniques to manage nocturnal worrying and anxious body states, like imagery work, breathing techniques and progressive muscular relaxation. However, with a long half-life, the duration of effect of promethazine at 10–19 h causes a hangover effect and is known to impede people being able to stick to a consistent rising time (a hugely important factor in regulating one's sleep pattern). In addition, use of sedative medication unhelpfully reinforces the idea that to be asleep is to be ‘knocked out’, anaesthetised, turned off like a computer – that is to say, impervious to external stimuli. During sleep, there is some maintenance of external awareness (for evolutionary survival reasons), yet people who are not ‘confident sleepers’ monitor their body and environment vigilantly, reinforcing a cycle of worries, anxious bodily feelings and unhelpful sleep–wake behaviours.

Second, anecdotally, colleagues suggest their preference for promethazine as a sleep aid over benzodiazepine receptor agonist medications (like Z-drugs) because it is ‘less addictive’. There are current active debates in psychiatry around what it is to be ‘addictive’ (see debates on the ‘addictive’ nature of selective serotonin reuptake inhibitors, for example), but many of the features of substance dependence are true for promethazine as they are for sedative hypnotics. Promethazine tolerance develops quickly, and there are well-documented withdrawal effects from regular use. Some long-term, off-label users of promethazine report not being able to stop their medication particularly because of rebound insomnia. Long-term use of promethazine as a sleep aid is anecdotally common, but national figures are not available, and neither are deprescribing guidelines for antihistamine agents. Importantly, there is plenty of recreational use of promethazine. Promethazine has a likeability for many users, and has a ‘street value’. It is also commonly used in combination with codeine, so nicknamed ‘lean’ or ‘purple drank’, because of promethazine's known effects in potentiating an opioid high, and research also evidences people self-medicating with promethazine to cope with undertreated mental health symptoms.^8^ Altogether, promethazine should be seen to be at least partially dependence-forming, and in the same ways as benzodiazepine receptor agonists.

Third, the side-effect profile of promethazine is particularly concerning for people using psychiatric services. These include QT prolongation, weight gain, nightmares, delirium and perhaps most importantly, significant anticholinergic side-effects, which also increase the risk of dementia.^9^ Herein, many other common medications prescribed for patients using psychiatric services, notably antipsychotics and other sedative psychotropics, have similar side-effect profiles that are compounded by promethazine. The effect on seizure threshold has shown mixed results in animal models. Patients should be told not to drive when using promethazine, and to be abstinent of other sedative drugs like alcohol and cannabis. Self-poisoning with promethazine in combination with other psychotropics appears to substantially increase fatality.^10,11^

As per NICE guidelines, CBTi is the first-line treatment for people with sleeping difficulties who are using psychiatric services, this can be delivered individually, in groups or online where facilitated CBTi is not available or is preferred by the patient. Mobile phone app-based, self-directed CBTi programmes are recommended by NICE, and are available with the National Health Service in some localities, although the evidence of its benefit for people with severe psychiatric co-morbidity is less robust. There should be screening for primary sleep disorders like sleep–wake phase disorders, restless leg syndrome and respiratory sleep disorders; reconciliation of existing psychiatric medication that might contribute to sleep difficulties; treatment of psychiatric causes of insomnia (substance misuse, psychosis, etc.) and referral to local specialist sleep services where needed. If medication is indicated because of limited response to CBTi, then it should be short term (<7 days). The evidence suggests that benzodiazepine receptor agonists offer a more effective intervention, with a side-effect profile of lesser concern, and do not produce a comparable hangover effect that impedes the employment of cognitive–behavioural techniques.^12^ Newer orexin antagonists are emergingly prescribed: daridorexant is now recently licensed in UK for patients who have not adequately responded to CBTi. Melatonin is licenced both as a hypnotic for insomnia in adults over 55 years old and for people with an intellectual disability, as well as treatment of certain sleep–wake disorders. Patients taking sedative hypnotics in the long term should be supported to gradually reduce their medication, under the supervision of specialist sleep services in some cases.

In short, we must reflect on our apparent increasing use of this 1950s medication, and hold it to the same standards that we would any new medication. When examined, we find that there is no good evidence for its use as a sleep aid, signs of active detriment and much better alternatives. Improving provision of CBTi for people using psychiatric services ought be a response to the increasing number of prescriptions. Promethazine likely has value as a short-term sedative agent for people in acute psychiatric crisis, but this should not be conflated with improving sleep quality.

## About the author

**Jacob D. King** is Honorary Clinical Research Fellow, Division of Psychiatry, Imperial College London, UK; and Psychiatry Resident, Central and North West London NHS Foundation Trust, London, UK.
